# CT Scanning and MATLAB Calculations for Preservation of Coptic Mural Paintings in Historic Egyptian Monasteries

**DOI:** 10.1038/s41598-019-40297-z

**Published:** 2019-03-07

**Authors:** Ahmed Sallam, Sayed Hemeda, Muhammet S. Toprak, Mamoun Muhammed, Moustapha Hassan, Abdusalam Uheida

**Affiliations:** 1 Faculty of Archaeology, Aswan University, Aswan, Egypt; 2 Department of Applied Physics, KTH-Royal Institute of Technology, SE-10691 Stockholm, Sweden; 3 Conservation Department, Faculty of Archaeology, Cairo University, 12613 Cairo, Egypt; 4 Department of Laboratory Medicine, Karolinska Institute, 14186 Huddinge, Stockholm Sweden

## Abstract

Investigations of Coptic mural paintings in historic churches and monasteries demand a deep understanding of the micro structure of the mural painting layers. The main objective of the present study is to study the efficiency of new avenues of computed X-ray tomography (CT Scan) and MATLAB in the analysis of Coptic mural paintings, either in the form of images or videos made to collect information about the physical characteristics of the material structure of the layers of mural paintings. These advanced techniques have been used in the investigation of samples of Coptic mural paintings dating back to the V–VIII century A.D, which have been collected from several locations in the Coptic monasteries in Upper Egypt. The application of CT-scanning is a powerful non-destructive tool for imaging and investigation which can be applied to the preservation of monuments made from many different materials. The second stage of research will be to characterize the materials through analytical techniques including XRD, XRF, EDX and FTIR to confirm the findings of CT scanning and to provide additional information concerning the materials used and their deterioration processes. This paper presents the results of the first pilot study in which CT scan and MATLAB have been utilized in combination for the non-destructive evaluation and investigation of Coptic mural paintings in Upper Egypt. The examinations have been carried out on mural painting samples from three important Coptic monasteries in Upper Egypt: the Qubbat Al Hawa Monastery in Aswan, the Saint Simeon Monastery in Aswan and the Saint Matthew the Potter Monastery in Luxor. This multi-stranded investigation has provided us with important information about the physical structure of the paintings, grains dimensions, grain texture, pore media characterization which include the micro porosity, BET and TPV, surface rendering, and calculation of the points in the surface through calculations completed using MATLAB. CT scanning assisted in the investigation and analyses of image surface details, and helped to visualize hidden micro structures that would otherwise be inaccessible due to over painting.

## Introduction

The digital preservation of historical monuments using advanced 3D measurement technologies such as CT scanning and MATLAB are becoming efficient tools for object mapping.

Samples from different periods and places can be scanned three-dimensionally (stereoscopically) using CT scanning. The specimen can be section cut at any angle without damaging the object, making it a very important tool for archaeological sampling. Moreover, the CT scans allow for the creation of a 3D structural image of the mural painting samples and enable investigation from any angle^1,2^.

The samples analysed here are the first from a Coptic mural painting to be examined by CT scanning. The results have yielded extensive information on the surfaces, porosities, and inclusions. CT scanning allows every type of defect to be seen, located in space, and measured^3,4^. This provides much more information than a single X-ray image and the evaluation quality is usually far better with CT than with digital X-ray. CT scanning is particularly useful for this study because it can help us to distinguish multi-layer overpainting on Coptic murals. Using this technique, we can observe hidden structures and employ cross sectioning to analyse overpainting and structure. Such advantages of this non-destructive technique are being exploited in increasingly imaginative ways across the cultural heritage sphere^1^.

Coptic mural paintings in the monasteries of Upper Egypt are fresco paintings. The technique of fresco painting consists of painting pigments onto a layer of plaster that is still wet or “fresh” as the Italian term fresco suggests. The fresco paintings under investigation here are either directly applied on plaster layers consisting of fresh lime, or onto plaster layers dating back to different periods (old layers) consisting of different minerals. The multi-layered structures of these overpainted murals are complex. Some of the monasteries examined in this project reveal important overpainted Coptic texts; CT scanning helped us to better understand this.

**Figure 1:**
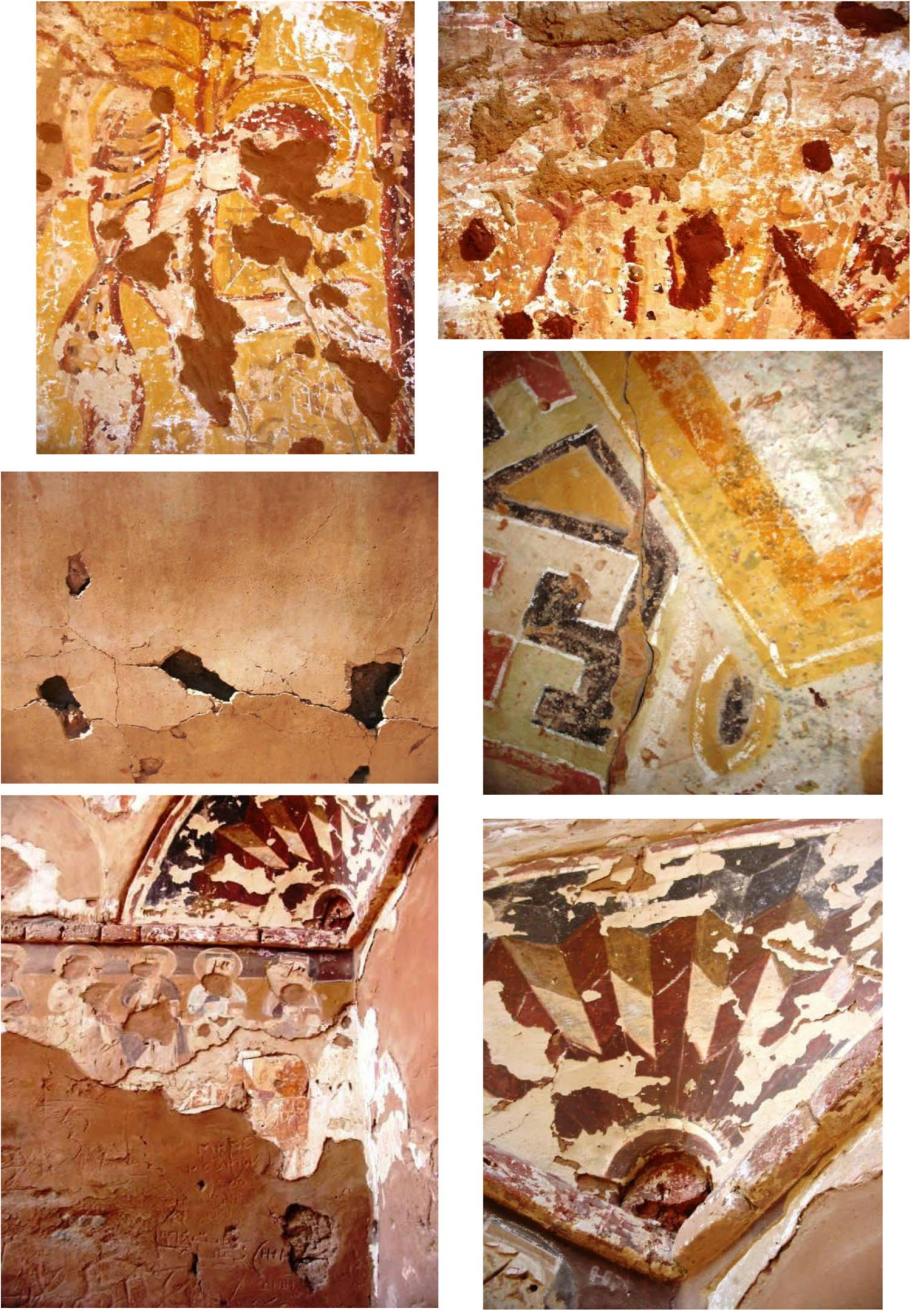
Deterioration of the mural painting at the St. Simeon monastery in Aswan. (**a,b**) Faulty restoration (**c,d**) crack formation in the real wall painting and holes, (**e**) plaster layers back to different periods (**f**) plaster detachment.

Considerable conservation work remains to be performed at these monasteries, for there are many more Coptic and Arabic texts preserved under a thin layer of plaster. These texts and the murals of the church are very important to the study of the history of Christianity and monasticism in Upper Egypt, and the paintings of the church provide invaluable documentation for the study of Coptic art. Unfortunately, the state of preservation for most of the murals bearing these texts is now very poor, (Fig. 1).

Investigation by CT scanning and examination by SEM-EDX all confirm the bad condition of these murals, which have been exposed to prolonged daily and seasonal variations of temperature, where in Aswan city, the temperature can reach 40–50 °C in the summer but can fall to 5 °C in the winter. Wind erosion, human handling, and fragmentation due to vandalism over the last forty years have also taken their toll; nevertheless, some parts of the fresco murals are still good quality and beautiful^5–8^.

The main objective of the present study is to study the efficiency of new avenues of computed X-ray tomography (CT scanning) and MATLAB in the analysis of Coptic mural paintings, regarding their physical characteristics and material structure in order to better understand their condition.

The second objective is to characterize the murals using analytical techniques such as EDX. This will help us to understand the materials involved, which is important both for the study of the ancient craftsmanship and the deterioration processes of the murals.

## Historical Context

### Qubbat Al-Hawa monastery in Aswan

**Figure 2:**
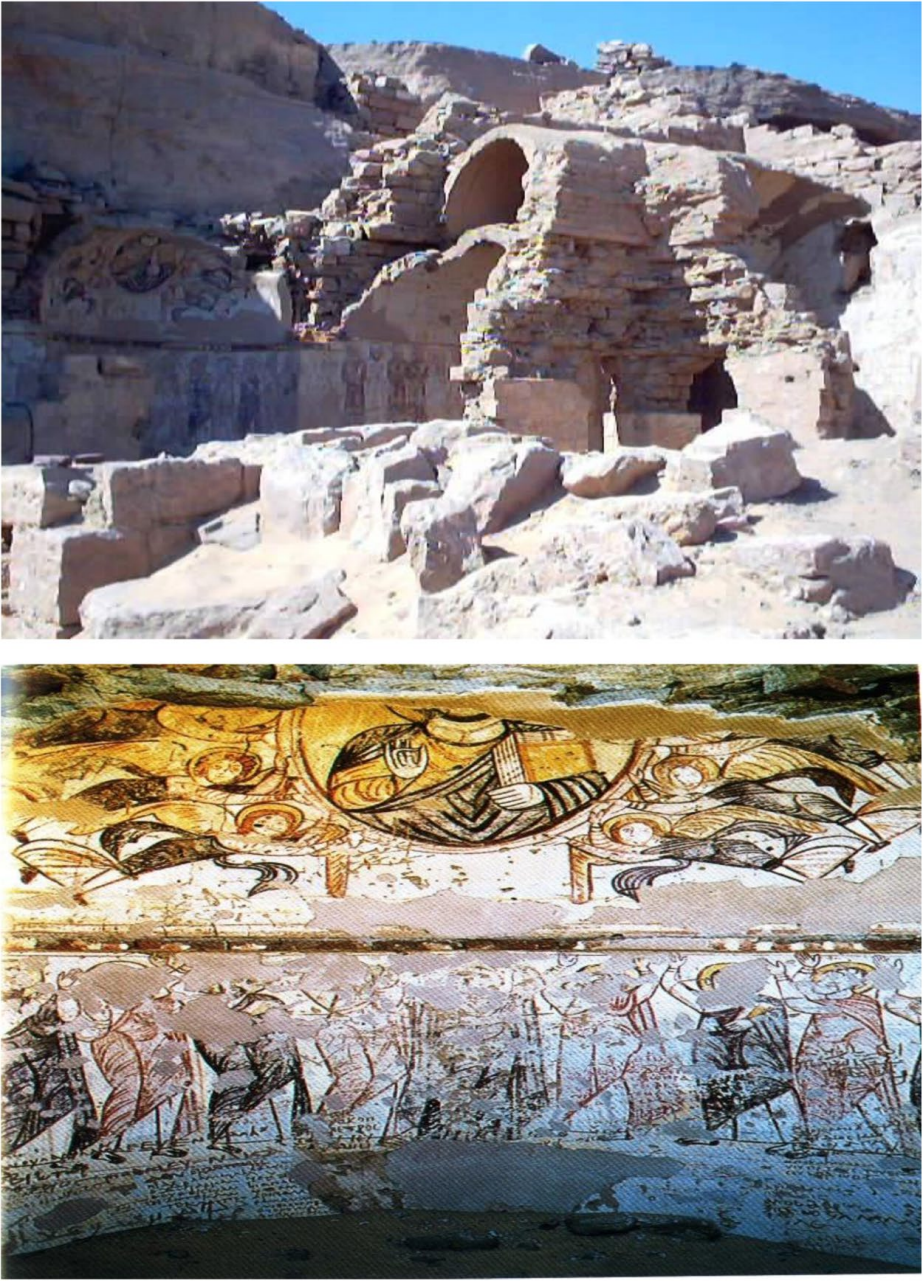
(**a**) The monastery of Qubbat Al-Hawa. (**b**) Lower part of an apse depicting the Virgin Mary flanked by the Apostles. The image shows exfoliation of the mural painting layer.

The Qubbat al-Hawa monastery is the second largest monastery in Egypt, which is located on the west bank of Aswan city. Today, this historic Coptic monastery is in ruins. It took its name from the hill where a shaykh is buried. On the flanks of this hill are the tombs of the governors of Aswan during the pharaonic New Kingdom. Qubbat Al-Hawa is located between the summit of the hill and the Nile River (Fig. 2). Church history records that it was probably built after the beginning of the Fatimid Period but before the end of the eleventh century. The golden age of the monastery was in the Fatimid period^5–8^.

### St. Simeon Monastery in Aswan

**Figure 3:**
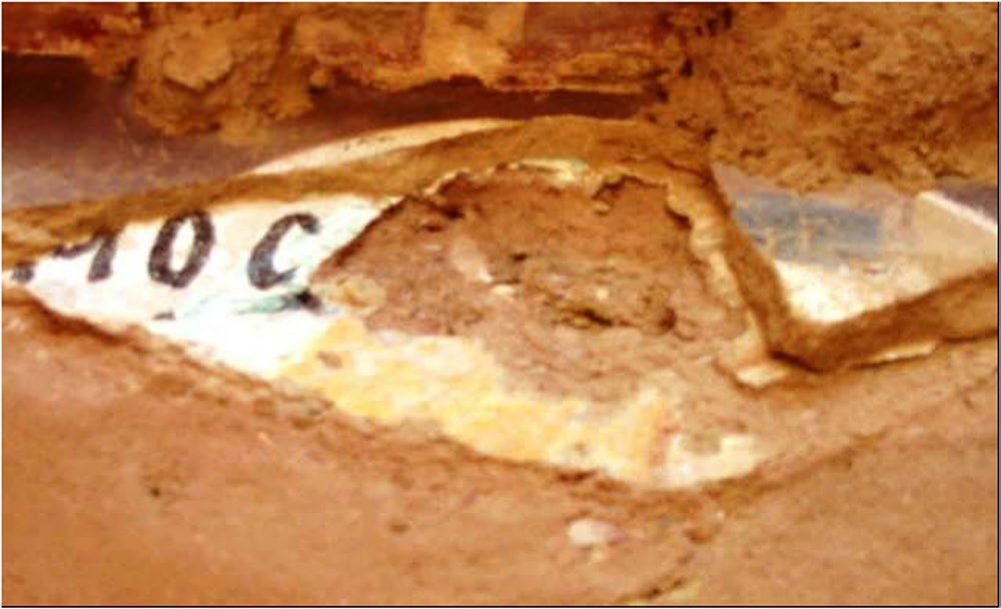
Detachments of multilayer (overpainting) back to different periods.

Although this monastery has been uninhabited since the twelfth century, it remains fairly well preserved. The St. Simeon monastery (also known as Deir Anba Sim’an, Monastery of Anba Hatre, Hidra, Hadri or Hadra) is still actively in use. The monastery was built during the first half of the eleventh century. By the end of the thirteenth century; it was one of the largest Coptic monasteries in Egypt (Fig. 3)^5,6,8^. The damage/deterioration of the Coptic mural paintings located in the Simeon monastery can be related to the following: 1. Loss of strength and granular disintegration due to chemical mineralogical physical changes, and convenient pigment detachment; 2. Loss of the thin painting applied to the subsurface layer, which has led to desegregation of the surface and diminished cohesion between the wall and the mortar of the fresco; 3. The alteration of the pigment, as a result of water penetration by diffusion through the fresco from the wet wall to the pictorial layer. As well as the anthropogenic actions and impacts^9–11^.

### Saint Matthew the Potter (Deir al-Fakhuri) in Esna - Luxor

**Figure 4:**
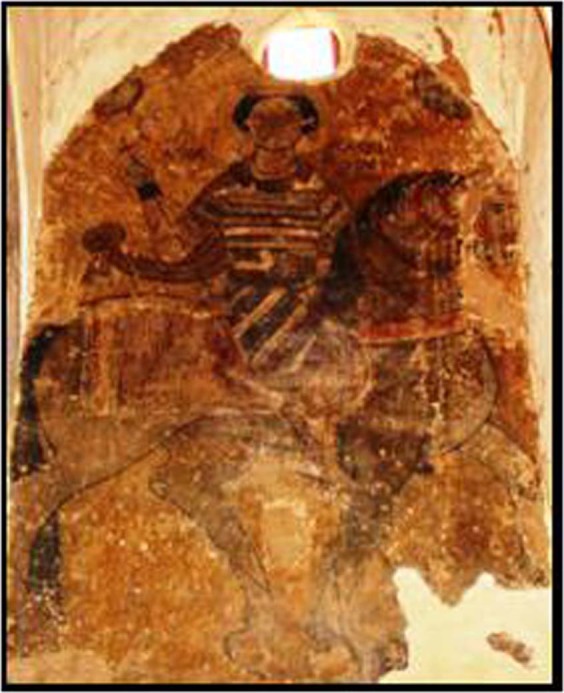
Detachments of parts of plaster.

The Saint Matthew the Potter Monastery is located at the far edge of the desert of Esna, about 6 miles (9 km) north of the city of Esna (in the Qena governorate), near to ancient Asphynis (present-day Asfun al-Mata’nah). The monastery is dates to the fifth and sixth centuries A.D. and was occupied by monks as recently as 1975. (Fig. 4)^6–8,12^.

## Materials and Experimental

### Sample collection

In this study we investigated three groups of samples; each group comprises 3 samples collected from different levels of mural paintings of three monasteries in Upper Egypt. The dimensions of the samples were variable, (the samples ranged from 1.5 cm × 0.8 cm × 0.5 cm to 2 cm × 1 cm × 0.9 cm), all samples were polished before the examination study.

**Figure 5:**
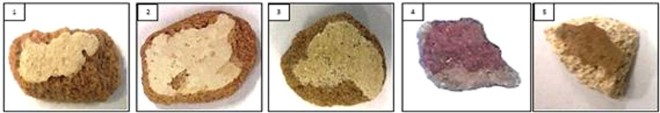
Images of the five samples collected from different sites (1)Monastery of Qubbat al-Hawa. (2) Monastery of Anba Hedra. (3) Monastery of St Matthew the Potter (Al– Fakhuri) near Esna.

The porosity of the samples was determined using MATLAB. The photographic images of the collected samples are show in Fig. 5, and details of the samples are listed in Table 1. All three monasteries under investigation are in a poor state of preservation; however, the mural paintings in the worst condition are those from the Monastery of Saint Simeon in Aswan. These frescoes have been partially lost due to granular disintegration resulting from chemical-mineralogical-physical changes. Pigments from the paintings had become detached due to temperature variations, so this sample was also examined using EDX.

**Table 1:** Details of the samples, typology and location.

Sample	Typology	Location
a. Monastery Qubbat al- Hawa	Coarse lower mud plaster layer	Aswan
b. Monastery St. Simeon	White mural painting	Aswan
c. Saint Matthew the Potter Monastery (Al - Fakhuri – Esna)	White mural painting	Luxor

### Method

#### CT scanning (imaging) and MATLAB porosity calculations

A CT system consists of a motorized X-ray source that rotates around a circular opening. All x-ray imaging is based on the absorption of X rays as they pass through the different parts of a sample. The information is picked up by detectors and transmitted to a computer to reconstruct all of the angles which are collected during one complete rotation. Samples were scanned and examined using a micro CT (Quantum FX, PerkinElmer, and Waltham, MA, USA). The scans were made under tube voltage 90 Kv, tube current 160 µA, Filed of View at 20 mm, scan time at 4.5 min. The CT scanning and examination of the mural samples was conducted at the Karolinska Institute, Stockholm, Sweden^4,13–16^.

#### Scanning Electron Microscopy-Energy Dispersive X-ray spectroscopy (SEM–EDX)

The morphology of fresco samples of the three monasteries was examined using Scanning Electron Microscopy attached with Energy Dispersive system (SEM, Gemini Zeiss-Ultra 55). EDX was used to determine the elemental composition of the coarse lower plaster layer. The cross sections of the samples were prepared from the small fragments taken from the mural painting surfaces. Samples were prepared and examined at KTH Department of Materials and Nano Physics, Stockholm, Sweden. For morphological characterization, mural paintings were scanned using a micro CT (Quantum FX, PerkinElmer, Waltham, MA, USA). The scans were made under tube voltage 90 kV, tube current 160 µA, field of view at 20 mm, and scan time of 4.5 min. Quantum FX is a cone-beam micro CT system, with the X-ray source and the detector rotated around a stationary sample by 360 degree during scanning. All X-ray imaging is based on the attenuation of X-ray as it passes through the different parts of a sample. The X-ray attenuation/co-efficient value based on the density difference between different parts of the sample is collected and constructed as CT images. By combining a series 2-D CT images generated from 360 degree rotation, a full 3-D image is reconstructed by built-in software Viewer (PerkinElmer). The special characteristics of the CT-images allow 3D and 2D reconstructions of the samples at a high defined resolution up to 4.5 µm. This allowed the study to distinguish the layer structure as well as to study morphology of the surfaces. All imaging was done at Karolinska Institute, Stockholm, Sweden.

## Results and Discussion

### CT Scan imaging results

The results details of the investigation by CT scan for the mural samples are observed and recorded; the significant results are summarized and classified according to the location of the samples as follow:

#### Monastery Qubbat al-Hawa in Aswan

The samples examined show that the painted layer was directly applied to the coarse lower plaster layer. The results also indicate that the plaster layers were applied in three layers in some parts of the monastery.

**Figure 6:**
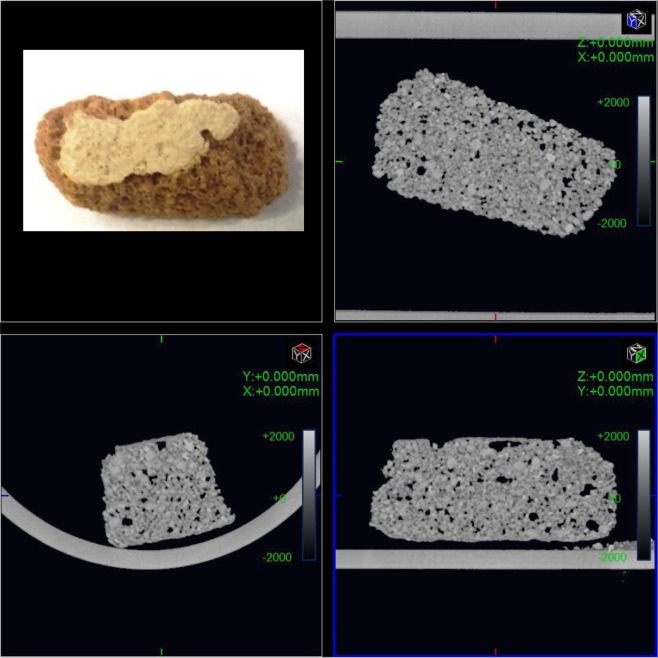
(**a**) CT image shows the Coptic mural painting at the Qubbat Al-Hawa Monastery in Aswan. (**b**) CT image of the sample structure showslarge porosity due to loss of minerals. (**c**) CT horizontal section showing overpainting (multi layers). (**d**) CT horizontal section showing detachment of pigments from the ground.

Investigation of the samples by CT scanning shows the loss of strength of the materials and granular disintegration due to the physicochemical actions, resulting in the loss of minerals,the average temperature in the selected sites varies from 50 °C in July to 10 °C in January. The high temperature may lead to thermal shock in the minerals and loss of cement material (calcite and gypsum), specifically gypsum (calcium sulphate dihydrate), which is dehydrated and transformed into a lower hydrous phase (hemihydrated phase), then finally transformed into the brittle anhydrous phase (anhydrite). The temperature in Aswan shifts dramatically from summer to winter, which causes the plaster to expand and contract repeatedly. Differing levels of expansion between the layers of the mural cause interlayer stress^17,18^ leading to the dislodging of the paint layer, as well as causing cracks, contraction fissuring and peeling of the vertical walls. The continual recurrence of these variations daily and seasonally makes the layers dislodge easily. The CT cross-section images revealed significant information on the hidden parts and overpainting of two layers. The first layer was found to be smooth and the second layer is a little thicker than the first, making it likely that the two layers come from different periods with different qualities and artistic styles as shown in Fig. 6c.

#### Monastery of St. Simeon (Anba Hedra) in Aswan

**Figure 7:**
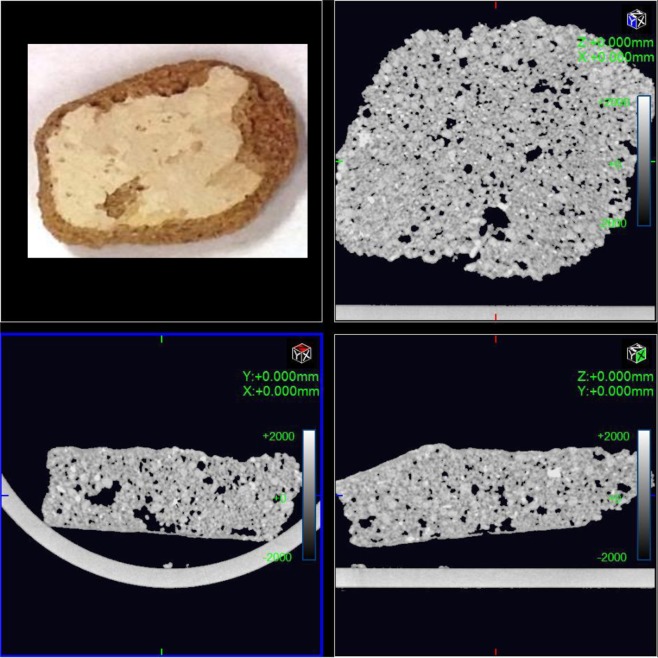
(**a**) CT image shows the Coptic mural painting from the St Simeon Monastery in Aswan. (**b**) CT of the horizontal section of the sample showing degradation of the surface, large porosity and loss of cohesion (powdering). (**c**) CT horizontal section showing flaking and cracked layers. (**d**) CT image showing two layers of the pigments on the top as well as the bottom degradation of the major composite (quartz) between grains, which leads with time to the loss of quartz grain, cracking and loss of plaster layers, in addition to the coarse lower plaster layer: a composite of quartz, clay minerals and calcite. Loss of quartz grains and clay minerals causes large holes, weak points and loss of the sample in the long term.

The characterization of mural samples was performed using CT scanning, as shown in Fig. 7. The samples have been characterized as having a type of surface defect on the plaster as indicated by the calculations completed in MATLAB.

**Figure 8:**
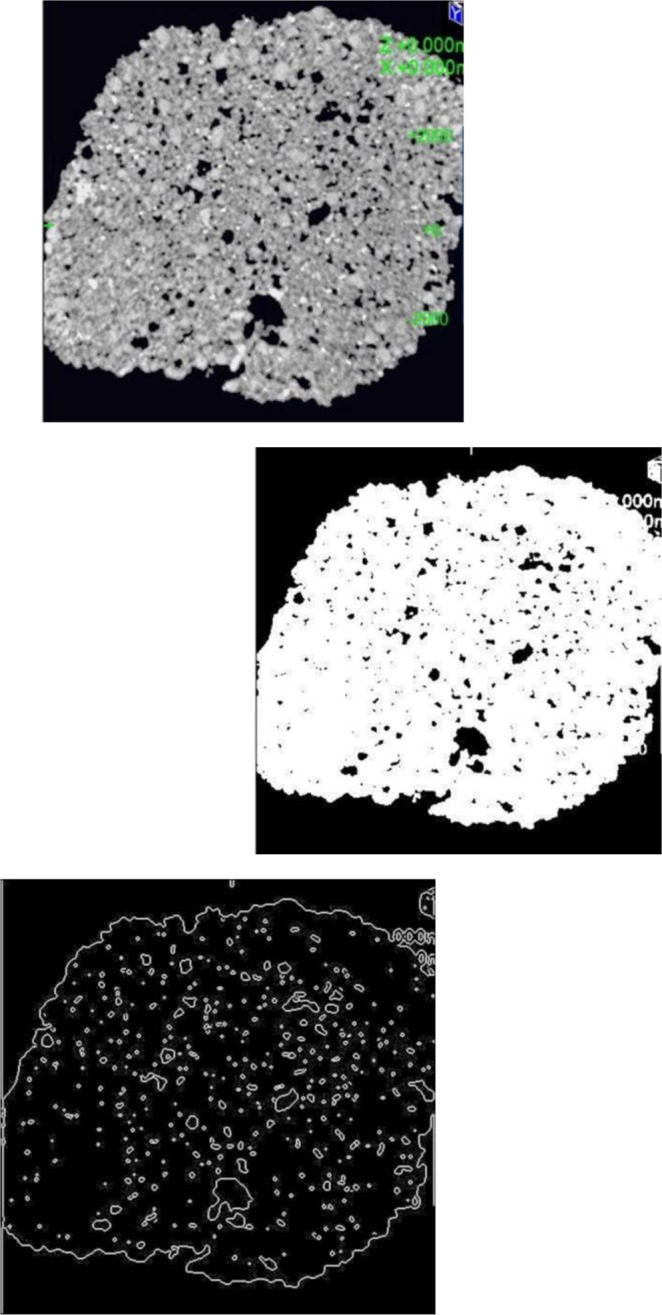
(**a**) Part one of the sample investigation by CT of the Coptic mural painting from the monastery of St Simeon in Aswan. (**b**) Black areas show porosity and gaps are due to the loss of binding mortar (**c**) CT image showing the surface. The black areas show porosity indicating the degradation of most of the surface and the major components.

The CT image shows a single slice illustrating the stratigraphy of a model fresco mural painting: two layers were combined in the first layer as a technique to produce good quality. These layers are the richest in quartz (as the major component) and clay minerals (Kaolinite and Orthoclase). The secondary layer is thinner than the first. In addition, some dark areas can be observed in the sample caused by the loss of quartz grains and clay minerals. The CT images show the detachment of the paint layer from the ground layer due to temperature and relative humidity variations, as well as the mechanical stresses and the different physicochemical properties of the samples because of different mineral content, as shown in Fig. 7. The combination of quartz, calcite (CaCo_3_) and clay minerals caused the change from gypsum to anhydrite, which is weaker than gypsum. This led to the eventual loss of quartz grains, cracks, loss of plaster layers and large porosity, finally this may lead to the flaking of the outer surface layer and loss of the original painting, as shown in Figs 7 and 8.

#### CT scan and MATLAB for Porosity Analysis and Pore Media Characterization

The new method called “The gray level method” which reflects the phenomenon of image processing and computation of ration between the volume of voids and the total volume of the entire sample.

The scan can show cross-sectional images of a specific area of the sample. A CT scanner emits a series of narrow beams through the sample as it moves through an arc. This is different from an X-ray machine, which sends just one radiation beam. The CT scan produces a more detailed final picture than an X-ray image. The CT scanner’s X-ray detector can see hundreds of different levels of density. This data is transmitted to a computer, which builds up a 3-D cross-sectional picture of different parts of the sample and displays it on the screen. The accuracy and speed of CT scans may be improved with the application of spiral CT, a relatively new technology. The beam takes a spiral path during the scanning, so it gathers continuous data with no gaps between images. Following from the fact that the density of the matter inside the sample is represented by the gray scale level, when X = gray level value (porous) and Y = number of pixels. CT scan images and MATLAB analysis allow the evaluation of porosity within the bulk volume of samples.

The computed tomography is based upon measuring of attenuated X-rays passed through a tested object along the set of defined paths followed by the reconstruction of an acquired dataset with the aid of mathematical reconstruction algorithms. The result represents the distribution of the attenuation coefficient μ(x, y) for individual pixels in each cross-section. The individual pixels are represented by CT numbers where the lowest CT number is set to black and the highest one to white. The measurement of empty space and the total volume can be expressed as the function. Taking into account the gray levels of CT image, so called a Digital terrain model could be created where the grey levels are related to the altitude from the ground or elevation. In this study, the method suggested by Taud *et al*.^19–23^ was considered. Taud, developed the grey level method based on Digital Terrain Model (DTM) theory. In this method, the CT image is symbolized as a Digital Terrain Model. The grey level in the DTM image is related to the altitude or elevation terrain. The porosity can be calculated as follows, Eq. (1):

1 $${\rm{\varphi }}(l)=\frac{{\sum }_{i=0}^{l}\,({\rm{l}}-{\rm{ri}})\,{\rm{H}}({\rm{ri}})}{l{\sum }_{i=0}^{l}\,{\rm{H}}({\rm{ri}})}$$

where Φ(l) is the pore distribution function, ri is a grey level related to the altitude, r H (ri) is the histogram of the image with grey levels in the range [r_min_, r_max_], r_min_ is the Minimum grey value, r_max_ is the maximum grey value and l is grey level in the range [r_min_, r_max_]^24^.

Figure 8 shows the Porosity and calculations in MATLAB, which is also detailed in Table 2.

**Table 2:** Percentage of surface which is porous: part one, part two and part three from one sample from the monastery of St. Simeon in Aswan.

Sample	Sample Porosity	Total Surface Porosity %
Part One	0.050	5%
Part Two	0.045	5%
Part three	0.078	8%
Average	0.058	6%

#### Part one of the samples

**Figure 9:**
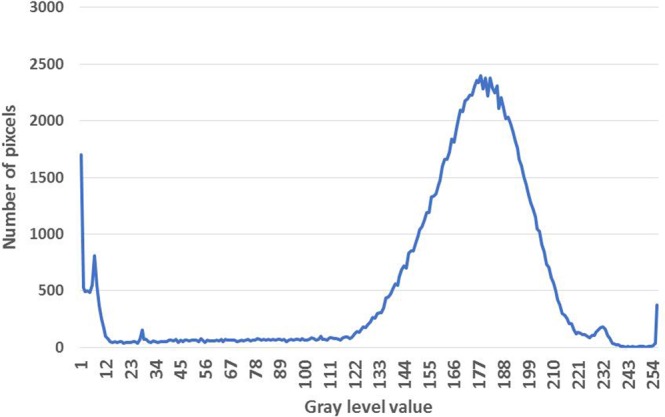
Plot of total surface porosity (part one).

The sample characterization was completed using CT scanning and the results are shown in detail in Fig. 8. The sample has been investigated to evaluate surface porosity and pore media characterization using MATLAB. The results indicate that the porosity is 0.050 of the total surface, this value signifies that 5% of the surface is porous, indicating a loss of around 5% of the composite minerals in the surface area. Figure 9 presents a plot of total surface porosity.

#### Part two of the sample

**Figure 10:**
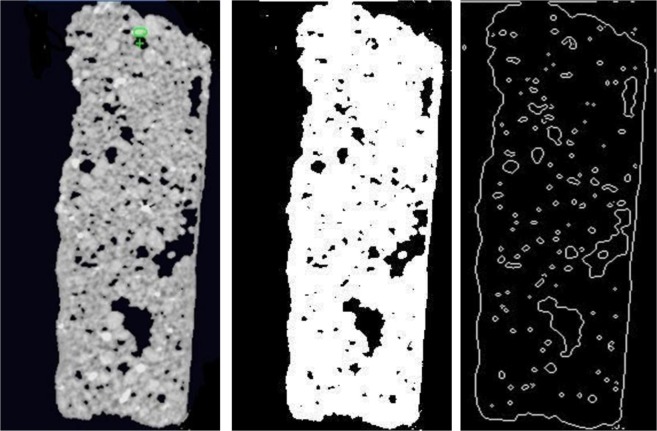
(**a**) Part two of the sample investigation by CT of the Coptic mural painting from the monastery of St Simeon in Aswan. (**b**) Black areas show porosity and contain gaps. CT image showing the surface as white areas indicating the loss of most surface area to porosity and deterioration of the major composites.

**Figure 11:**
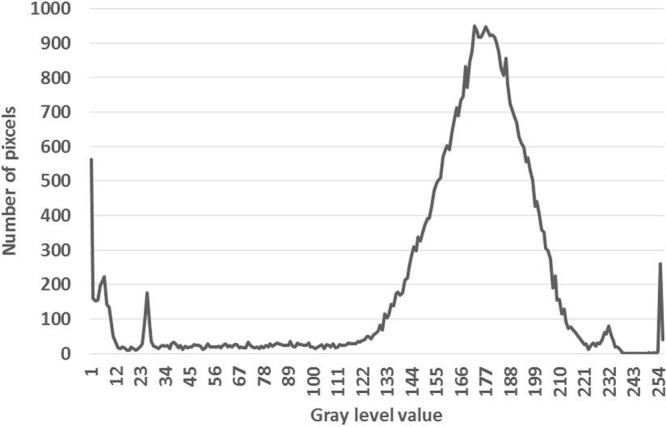
Plot of total surface porosity (part two).

The results of the CT scanning are shown in Fig. 10. The MATLAB calculations show that the sample has a porosity of 0.045 of the total surface. This value indicates that the surface porosity is high and there is a loss of around 5% of the composite minerals. Figure 11 shows a plot of the porosity of the total surface. CT scan images and MATLAB analysis allow the evaluation of porosity within the bulk volume of samples.

#### Part three of the sample

**Figure 12:**
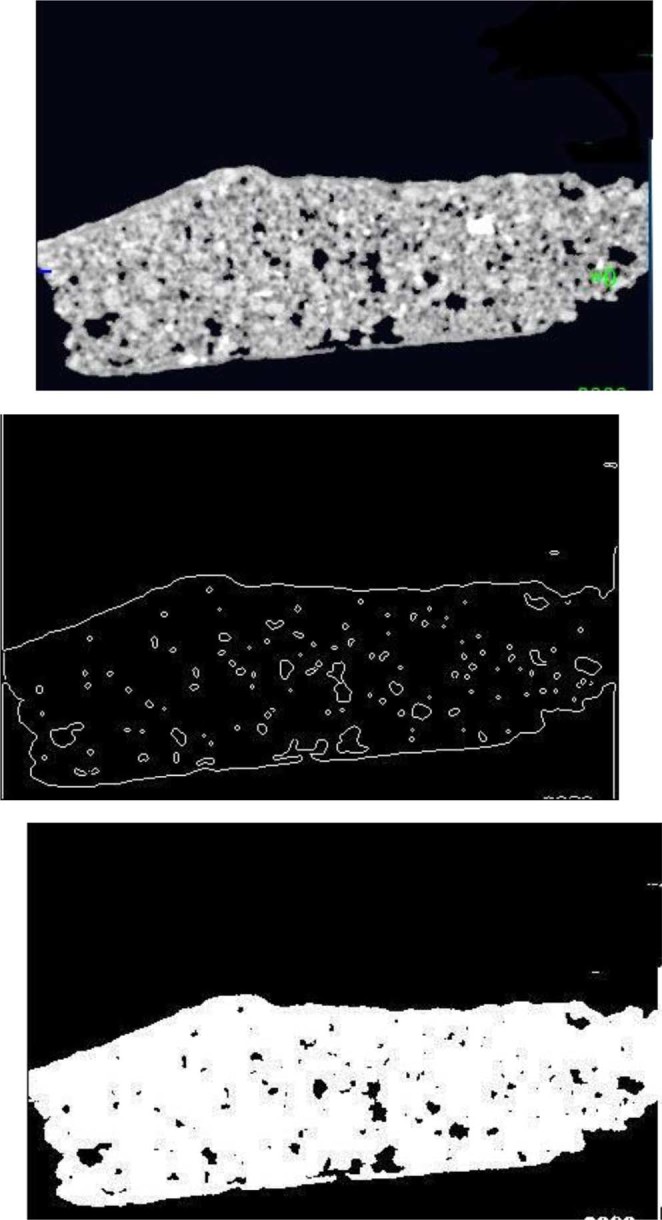
(**a**) Part three of the sample investigation by CT of the Coptic mural painting from the monastery of St Simeon in Aswan). (**b**)Black areas show porosity and contain gaps. CT image showing the surface as white areas with porosity areas in black indicating the loss of most of the surface and the major composites.

**Figure 13:**
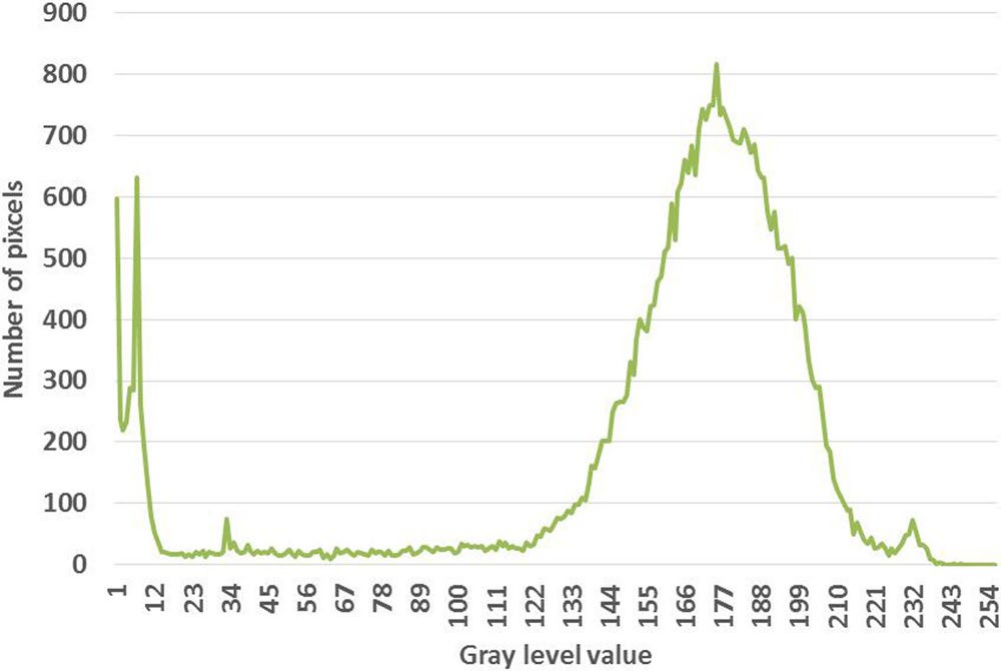
Plot of total surface porosity (part three).

Figure 12 shows the images from CT scanning. The sample has been characterized using MATLAB which indicated that the porosity is 0.078 of the total surface; this value indicates a loss of around 8% of the mineral composite. Figure 13 shows a plot of the porosity the total surface. The results from all three parts are summarized in Table 2.

By calculating together these three cross sections of the sample, we find an average porosity value of 0.058, which means that there has been loss of around 6% of the composite minerals. The monastery is in a bad condition and the fresco murals need immediate conservation and consolidation.

#### Al Fakhuri Monastery – Esna

**Figure 14:**
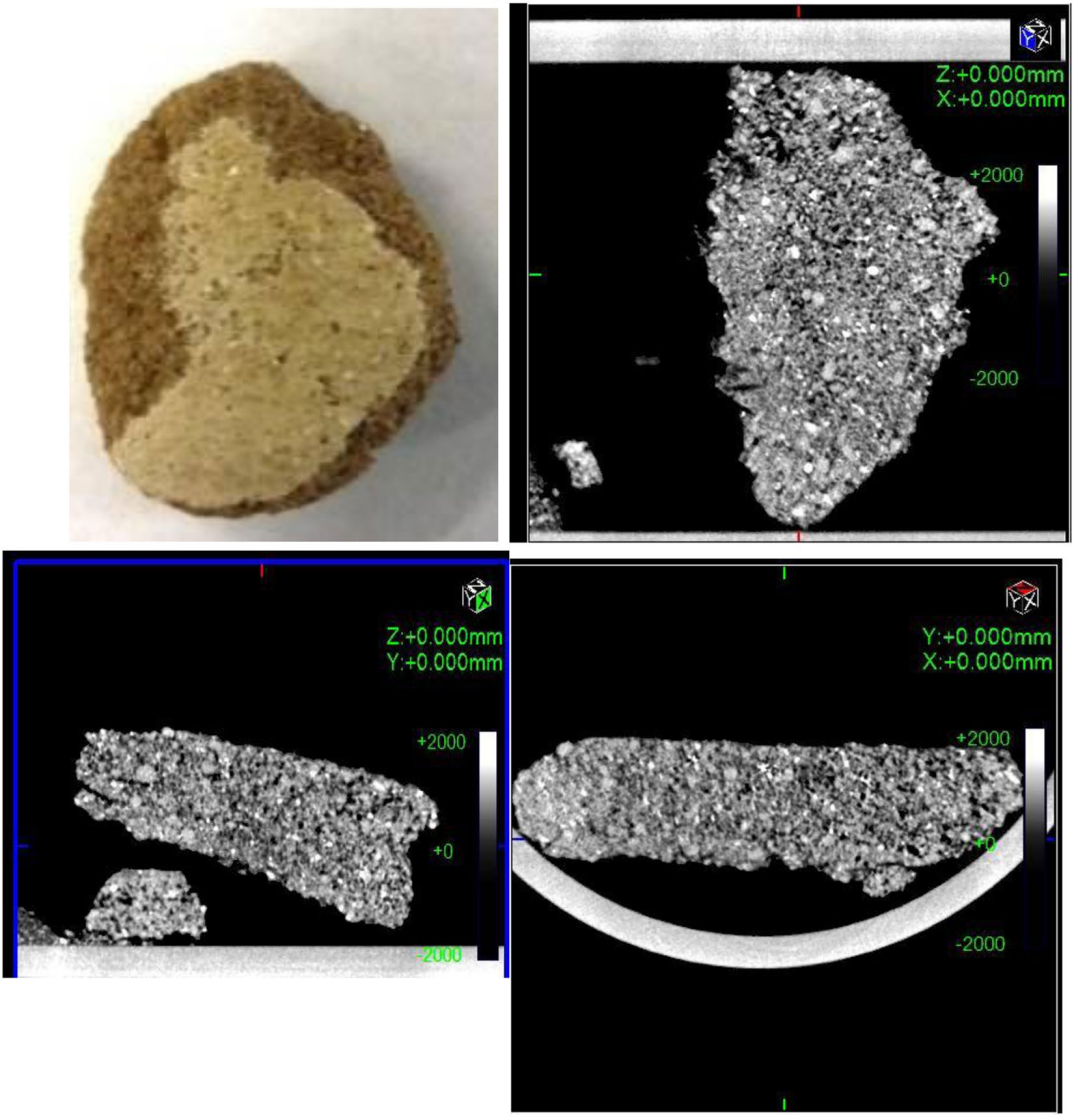
(**a**) Three orthogonal views of the mural painting from the monastery Matthew the Potter (Esna –Luxor) required for obtaining morphometric measurements. (**b**) CT horizontal section of the sample showing degradation of the surface, porosity and white areas are calcite and gypsum. (**c**) CT horizontal section showing the paint layer starting to flake and visible layer cracking. (**d**) CT showing degradation of the major composite (quartz) between grains leading in time to loss of quartz grain, cracks and loss of plaster layer.

The CT image (Fig. 14) of the sample from the Al Fakhuri Monastery shows that the painted layer has been applied directly on top of the coarse lower plaster layer. The plaster in this monastery was applied in one layer, which was the surface layer. The sample has been characterized to yield the grains dimensions in the surface and shape.

The cross-section stratigraphy of the painted layer reveals it to be a single composite layer. The gray areas are clay minerals, the white areas are quartz, calcite and gypsum, but the black areas are loss of composite minerals.

### Energy Dispersive X-Ray Analysis (EDX)

**Figure 15:**
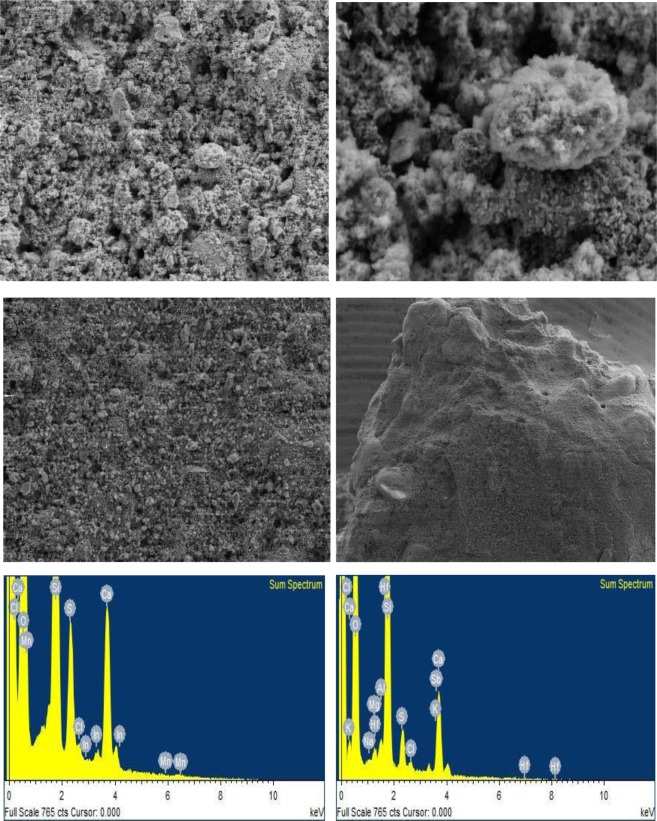
(**a–d**) SEM showing morphology of the coarse lower plaster layer of the St Simeon monastery, (**e,f**) EDX spectrum of a small area taken from the coarse lower plaster layer of mural at the monastery of St Simeon in Aswan.

EDX micro elemental analysis was conducted on the coarse lower plaster layer of the fresco murals from the monastery of St. Simeon in Aswan. The results showed the presence of different elements sodium (Na), manganese (Mn), magnesium (Mg), aluminium (Al), silica (Si), sulphur (S), chloride (Cl), and calcium (Ca), which are presented in Fig. 15e,f and are summarized in Table 3.

**Table 3:** EDX composition in wt. for coarse lower plaster layer of monastery Anba Hedra in Aswan.

Element	O	Na	Mg	Al	Si	S	Cl	K	Ca	Sb	Hf	C	Total
Weight%	45.4	0.2	0.4	1	16.8	2.6	0.7	0.7	10.5	2.3	2.5	17.8	100

## Conclusion

CT scanning is well-suited to artefacts and cultural material documentation via high-resolution imagery, allowing thorough investigation of the samples and objects from different angles. The results of CT scanning in this study strengthen the case for the use of this technology in the examination, documentation and conservation of Coptic mural paintings. We highlight the following points as significant results of our research: the three parts of the sample from the St. Simeon Monastery were investigated using CT images and MATLAB porosity calculations; the sample had 0.05803 mean porosity content, corresponding to a loss of around 5.8% of the composite minerals. This is a significant level of loss indicating that the paintings in this monastery need to be quickly conserved and consolidated. Most of the CT images show the detachment of the paint layer and separation from the plaster ground layer. This may have been caused by fluctuations of temperature and relative humidity through the days and seasons, also may it is attributed to the mechanical stresses and the differential physic-chemical properties of the minerals due to the difference in sample mineral composition, according to the result obtained from CT images and micro analysis by EDX. The EDX results show the major elemental composition of the coarse lower plaster layer, silica (Si) and calcite (Ca) was identified as the main or major elements of the paint layer in semi fresco technique.

The results of this pilot study have demonstrated the value of CT scanning and porosity calculations for providing detailed information regarding the state of preservation of the mural paintings found in the Coptic monasteries of Upper Egypt. The next stage of the project will build upon these findings to design an appropriate strengthening and retrofitting intervention plans and strategies for this unique culture heritage.
